# Glucose Metabolism Disorder Is Associated with Pulmonary Tuberculosis in Individuals with Respiratory Symptoms from Brazil

**DOI:** 10.1371/journal.pone.0153590

**Published:** 2016-04-14

**Authors:** Jilson L. Almeida-Junior, Leonardo Gil-Santana, Carolina A. M. Oliveira, Simone Castro, Aparecida S. Cafezeiro, Carla Daltro, Eduardo M. Netto, Hardy Kornfeld, Bruno B. Andrade

**Affiliations:** 1 Unidade de Medicina Investigativa (UMI), Laboratório Integrado de Microbiologia e Imunorregulação (LIMI), Centro de Pesquisas Gonçalo Moniz (CPqGM), Fundação Oswaldo Cruz (FIOCRUZ), Salvador, Bahia, 40296-710, Brazil; 2 Curso de Medicina, Faculdade de Tecnologia e Ciências (FTC), Salvador, Bahia, 41741-590, Brazil; 3 Multinational Organization Network Sponsoring Translational and Epidemiological Research (MONSTER) Initiative, Fundação José Silveira, Salvador, Bahia, 40210-320, Brazil; 4 Centro de Pesquisa, Instituto Brasileiro para Investigação da Tuberculose (IBIT), Fundação José Silveira, Salvador, Bahia, 40210-320, Brazil; 5 Hospital Universitário Professor Edgard Santos, Universidade Federal da Bahia, Salvador, Bahia, 40110-060, Brazil; 6 Department of Medicine, University of Massachusetts Medical School, Worcester, Massachusetts, 01655, United States of America; University of Cape Town, SOUTH AFRICA

## Abstract

**Background:**

Diabetes mellitus (DM) has been associated with increased risk for pulmonary tuberculosis (PTB) in endemic settings but it is unknown whether PTB risk is also increased by pre-DM. Here, we prospectively examined the association between glucose metabolism disorder (GMD) and PTB in patients with respiratory symptoms at a tuberculosis primary care reference center in Brazil.

**Methods:**

Oral glucose tolerance test was performed and levels of fasting plasma glucose and glycohemoglobin (HbA1c) were measured in a cohort of 892 individuals presenting with respiratory symptoms of more than two weeks duration. Patients were also tested for PTB with sputum cultures. Prevalence of pre-DM and DM (based on HbA1c) was estimated and tested for association with incident PTB. Other TB risk factors including smoking history were analyzed.

**Results:**

The majority of the study population (63.1%) exhibited GMD based on HbA1c ≥5.7%. Patients with GMD had higher prevalence of PTB compared to normoglycemic patients. Individuals with DM exhibited increased frequency of TB-related symptoms and detection of acid-fast bacilli in sputum smears. Among patients with previous DM diagnosis, sustained hyperglycemia (HbA1c ≥7.0%) was associated with increased TB prevalence. Smoking history alone was not significantly associated with TB in our study population but the combination of smoking and HbA1c ≥7.0% was associated with 6 times higher odds for PTB.

**Conclusions:**

Sustained hyperglycemia and pre-DM are independently associated with active PTB. This evidence raises the question whether improving glycemic control in diabetic TB patients would reduce the risk of TB transmission and simultaneously reduce the clinical burden of disease. A better understanding of mechanisms underlying these associations, especially those suggesting that pre-DM may be a factor driving susceptibility to TB is warranted.

## Introduction

Tuberculosis (TB) remains a major global health problem causing 9.6 million new cases and 1.5 million deaths in 2014 [1]. Adequate TB control at the population level requires better understanding of interactions with potential comorbid risk factors. The most significant acquired TB risks factors are undernutrition, smoking, AIDS, and DM [2–5]. These comorbidities increase the risk to develop active TB and the risk for adverse outcomes. Globally, 15% of TB cases are estimated to be attributable to DM [6]. The number of people worldwide with DM is expected to rise by 55% during the next 20 years, with the largest increase in countries where TB is already endemic [7]. At present, seven of the 10 countries with the highest number of DM cases also have a high TB burden [8,9]. Rising DM prevalence presents challenges in clinical TB management since it is associated with higher risk for adverse outcomes and relapse after the initiation of antituberculous treatment (ATT) [10–13]. In the coming years, DM will have an increasingly important detrimental effect on global TB elimination.

It is imperative to develop a better understanding of the interactions between GMD and TB, which may differ in a population-specific manner. Brazil is among the 22 countries identified by the World Health Organization (WHO) that concentrate 82% of TB cases worldwide [1]. Brazil also has the highest number of DM cases within the South and Central America, totaling 12.4 million individuals [6]. Few studies have investigated the interaction between these conditions systematically in this country [11,14]. In the present study, we performed an investigation of DM and TB in patients presenting with respiratory symptoms to a TB reference center in a highly endemic area in Northeast Brazil. Our results revealed a previously unappreciated high prevalence of GMD in individuals with respiratory symptoms and confirmed the previously reported association between established DM and TB. The data also suggest that pre-DM may also substantially increase the odds for TB disease.

## Methods

### Ethics statement

This study was conducted according to the principles expressed in the Declaration of Helsinki and was approved by the Ethics Committee of the Maternidade Climério de Oliveira, Federal University of Bahia (CAAE—0115.0.054.000–09). Written informed consent was obtained from all participants.

### Study design

The study was performed at the Instituto Brasileiro para a Investigação da Tuberculose (IBIT, Brazilian Institute for TB investigation). This philanthropic TB primary care reference center annually treats 10–15% TB cases of the city of Salvador, Bahia [15]. Diagnosis of TB at IBIT follows the guidelines of the Brazilian Society of Pulmonology and Tisiology [16], which is similar to WHO recommendations [17]. For the present study, PTB diagnosis was performed as the following: three sputum smears were examined by fluorescence microscopy, processed by the modified Petroff’s method and cultured on Lowenstein-Jensen medium. The present study did not perform screening for latent TB infection. At the time of this study, all identifying patient information was either coded or redacted by IBIT. All materials given to the research team were de-identified.

We prospectively investigated a cohort of adult patients (≥18 years old) with respiratory symptoms who sought care at IBIT. The initial population consisted of all symptomatic adult patients examined between May, 2010, and September, 2011 (n = 1,545). Symptomatic patients were defined by the presence of cough for ≥2 weeks in addition to at least one of the following: fever, night sweats, malaise, weight loss, hemoptysis or dyspnea. Exclusion criteria were age <18 years, previous diagnosis/treatment of TB, self-reported pregnancy, presence of psychiatric disease that might hamper proper application of the enrollment questionnaire. All patients were seen by nurses and physicians from IBIT at the time of initial evaluation. Of these, 192 individuals declined participation and 372 were excluded based on the study criteria. After signing the informed consent, a standardized questionnaire about demographic characteristics, medical history, and health-related habits was applied.

**Fig 1:**
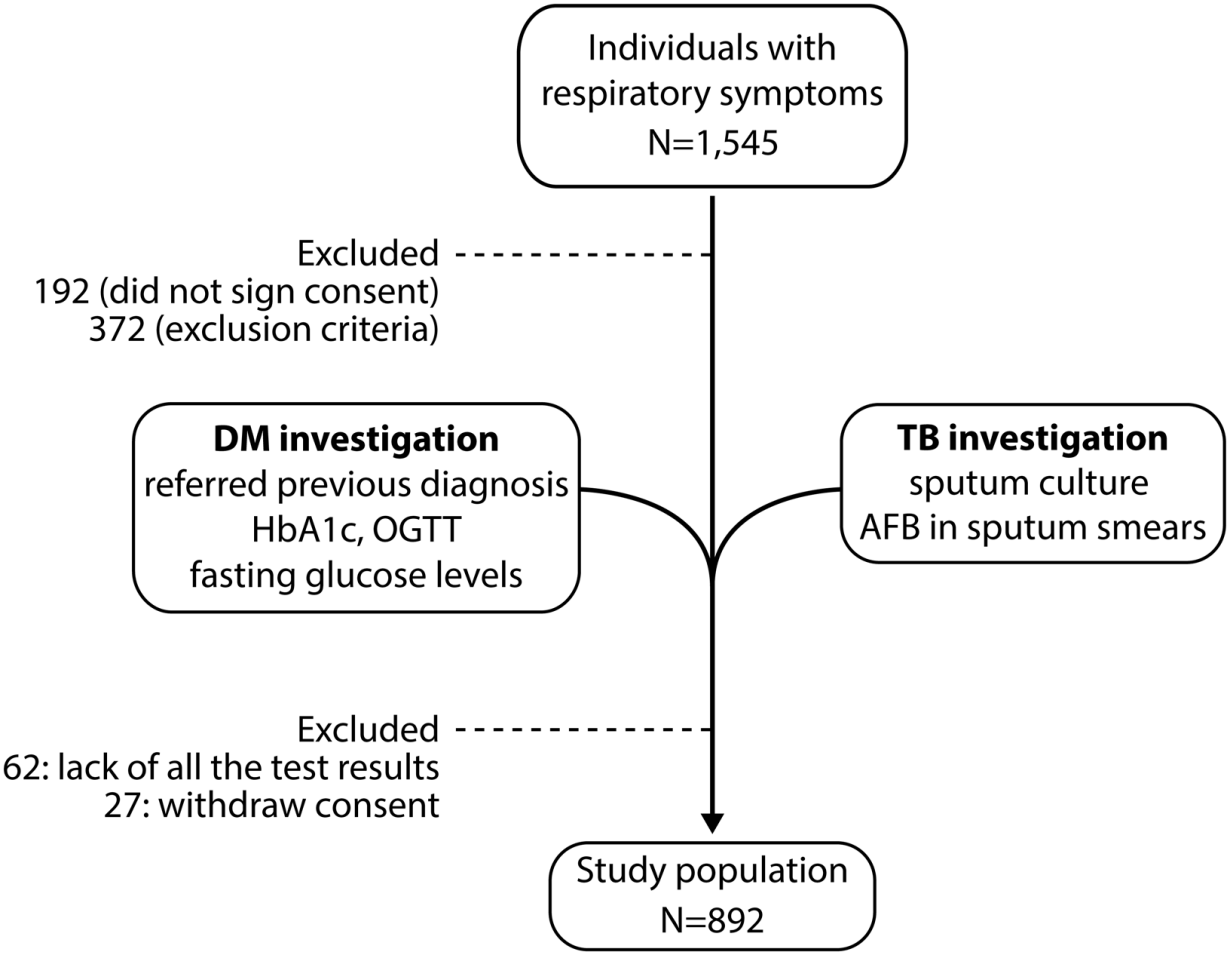
Overall study design. Individuals presenting with respiratory symptoms (n = 1,545) were recruited at a TB primary care reference center in Brazil. In two consecutive visits (1–3 days apart), all individuals were screened for diabetes / glucose metabolism disorder in the clinical interview and with laboratory tests (glycated hemoglobin, HbA1c; oral glucose tolerance test, OGTT; fasting glucose levels). Individuals were simultaneously tested for pulmonary TB by identification of acid-fast bacilli (AFB) in sputum smears and positive sputum culture for *Mycobacterium tuberculosis*. Details of the study design and patient population are described in Methods.

Random capillary glucose levels were measured on initial screening. Patients returned the following day, when blood samples were obtained after an overnight fast (12 h) and tested with an automatic clinical chemistry analyzer (Hitachi, LABOSPECT 008, Tokyo, Japan). HbA1c was assessed by high-performance liquid chromatography using a method certified by the National Glycohemoglobin Standardization Program. All patients also underwent a 75-g challenge oral glucose tolerance test (OGTT). The presence of DM was defined in accordance with American Diabetes Association (ADA) guidelines as 2-h glucose ≥11.1 mmol/L, HbA1c ≥6.5% or fasting plasma glucose ≥7.0 mmol/L [18]. In patients reporting a prior history of DM, HbA1c ≥7.0% was used to define uncontrolled DM. Pre-DM was defined by HbA1c 5.7–6.4%. In the present study, we define glucose metabolism disorder (GMD) as the presence of DM or pre-DM according to ADA guidelines. An additional 89 patients were excluded from the analyses because they either missed laboratory tests/results (n = 62) or had withdrawn consent after lab tests were performed (n = 27). After all the procedures, a total of 892 individuals were included in our analyses. The overall study design is illustrated in Fig 1.

### Data analysis

Median values with interquartile ranges (IQR) were used as measures of central tendency. The Fisher’s (two groups) or chi-square (three groups) tests were used to compare frequency data (nominal variables). Continuous variables were compared between the study groups using the Mann-Whitney test. Binary logistic regression analysis was used to test associations between increasing levels of HbA1c, OGTT or fasting glucose values and the odds for PTB. Multinomial logistic regression analyses adjusted for age, gender and body mass index (BMI) were performed to assess the odds ratios (OR) of the associations between presence of DM, uncontrolled DM and/or smoking history and TB diagnosis. During the development of the statistical methods, the regression models employed were validated using bootstrap (repeated 200 times). A p-value below 0.05 was considered statistically significant. The statistical analyses were performed using SPSS 20.0 (IBM statistics), Graphpad Prism 6.0 (GraphPad Software, San Diego, CA) and JMP 11.0 (SAS, Cary, NC, USA).

## Results

The median age of patients (n = 892) was 49 years (IQR: 38–58), the majority female (55.3%) and median BMI was 22.6 kg/m^2^ (IQR: 19.8–26.2) (Table 1). Regarding lifestyle habits, 4.6% of patients reported chronic alcoholism, 42.4% smoking history and 2.4% used illicit drugs. The prevalence of PTB in the study population was 11.8% (n = 105). The most frequent documented comorbidities aside from TB were chronic obstructive pulmonary disease (COPD, 3.8%), HIV/AIDS (1.2%), pulmonary silicosis (2.5%) and cancer (1.1%) (Table 1). Patients who were excluded from the study due to lack of test results and from whom we had authorization to keep primary data (n = 62) were on average younger (median 43, IQR: 32.3–52.5 vs. 49, 38–58; p = 0.007) and more frequently reported use of illicit drugs (9.7% vs. 2.4%; p = 0.005) than those included in the additional analyses. These two groups of patients were similar with regard to gender (p = 0.693), smoking (p = 0.351), chronic alcoholism (p = 0.214) and comorbidities such as PTB (p = 0.299), HIV (p = 0.555), cancer (p = 1.0), COPD (p = 0.728) and silicosis (p = 0.391) (Table 1). Patients diagnosed with PTB at the admission were more frequently male (54.3%) and were on average younger, exhibited lower BMI values and reported more frequently chronic alcoholism compared to non-PTB individuals (Table 2). Smoking history and frequency of comorbidities were similar between PTB and non-PTB patients (Table 2). Within the respiratory symptoms evaluated, PTB patients exhibited higher frequency of fever, night sweats and weight loss compared with non-PTB subpopulation (Table 2).

**Table 1:** Characteristics of the Study Population. For more information in study design, see Methods and Fig 1. Frequency data were compared using the Fisher’s exact test and continuous variables (age and BMI) were compared using the Mann-Whitney test. BMI, body mass index; COPD, chronic obstructive pulmonary disease; IQR, interquartile range.

Characteristic	Patients with respiratory symptoms	Excluded Patients	P-value
	N = 892	N = 62	
**Female—no. (%)**	493 (55.3)	36 (58.1)	0.693
**Median Age—y (IQR)**	49 (38–58)	43 (32.3–52.5)	0.007
**Median BMI—kg/m^2^ (IQR)**	22.6 (19.8–26.2)	22.2 (19.9–24.9)	0.225
**Lifestyle habits—no. (%)**			
Chronic Alcoholism	41 (4.6)	5 (8.1)	0.214
Smoking	378 (42.4)	22 (35.5)	0.351
Illicit drugs use	21 (2.4)	6 (9.7)	0.005
**Comorbidity—no. (%)**			
Pulmonary TB	105 (11.8)	4 (6.4)	0.299
HIV/AIDS	11 (1.2)	1 (1.6)	0.555
Cancer	10 (1.1)	0 (0)	1.000
COPD	34 (3.8)	3 (4.8)	0.728
Silicosis	22 (2.5)	0 (0)	0.391

**Table 2:** Characteristics of PTB and non-TB patients. Patients with respiratory symptoms (n = 892) were screened for pulmonary tuberculosis (PTB) and classified as non-PTB or PTB according to culture positivity. Frequency data were compared using the Fisher’s exact test and continuous variables (age and BMI) were compared using the Mann-Whitney test. See Table 1 for abbreviations.

Characteristic	(non-PTB / PTB)	non-PTB	PTB	P-value
		n = 787	n = 105	
**Female—no. (%)**	787 /105	445 (56.5)	48 (45.7)	0.037
**Median Age—y (IQR)**	787 /105	50 (38–59)	45 (30–55)	0.0004
**Median BMI—Kg/m**^**2**^ **(IQR)**	775 /105	23.1 (20.2–26.7)	20.0 (18.3–22.7)	<0.0001
**Lifestyle habits—no. (%)**				
Chronic Alcoholism	787 /105	30 (3.8)	11 (10.5)	0.005
Smoking	787 /105	339 (43.0)	39 (37.1)	0.293
**Comorbidity—no. (%)**				
HIV/AIDS	787 /105	11 (1.4)	0 (0)	0.628
Cancer	787 /105	10 (1.3)	0 (0)	0.616
COPD	787 /105	32 (4.1)	2 (1.9)	0.415
Silicosis	787 /105	20 (2.5)	2 (1.9)	1.000
**Clinical symptoms—no. (%)**				
Cough	787 /105	750 (95.3)	103 (98.1)	0.304
Fever	787 /105	415 (52.7)	68 (64.8)	0.021
Night sweats	787 /105	411 (52.2)	70 (66.7)	0.006
Malaise	787 /105	581 (73.8)	75 (71.4)	0.637
Weight loss	787 /105	468 (59.5)	87 (82.8)	<0.0001
Hemoptysis	787 /105	160 (20.3)	21 (20.0)	1.000
Dyspnea	787 /105	487 (61.9)	61 (58.1)	0.456

**Fig 2:**
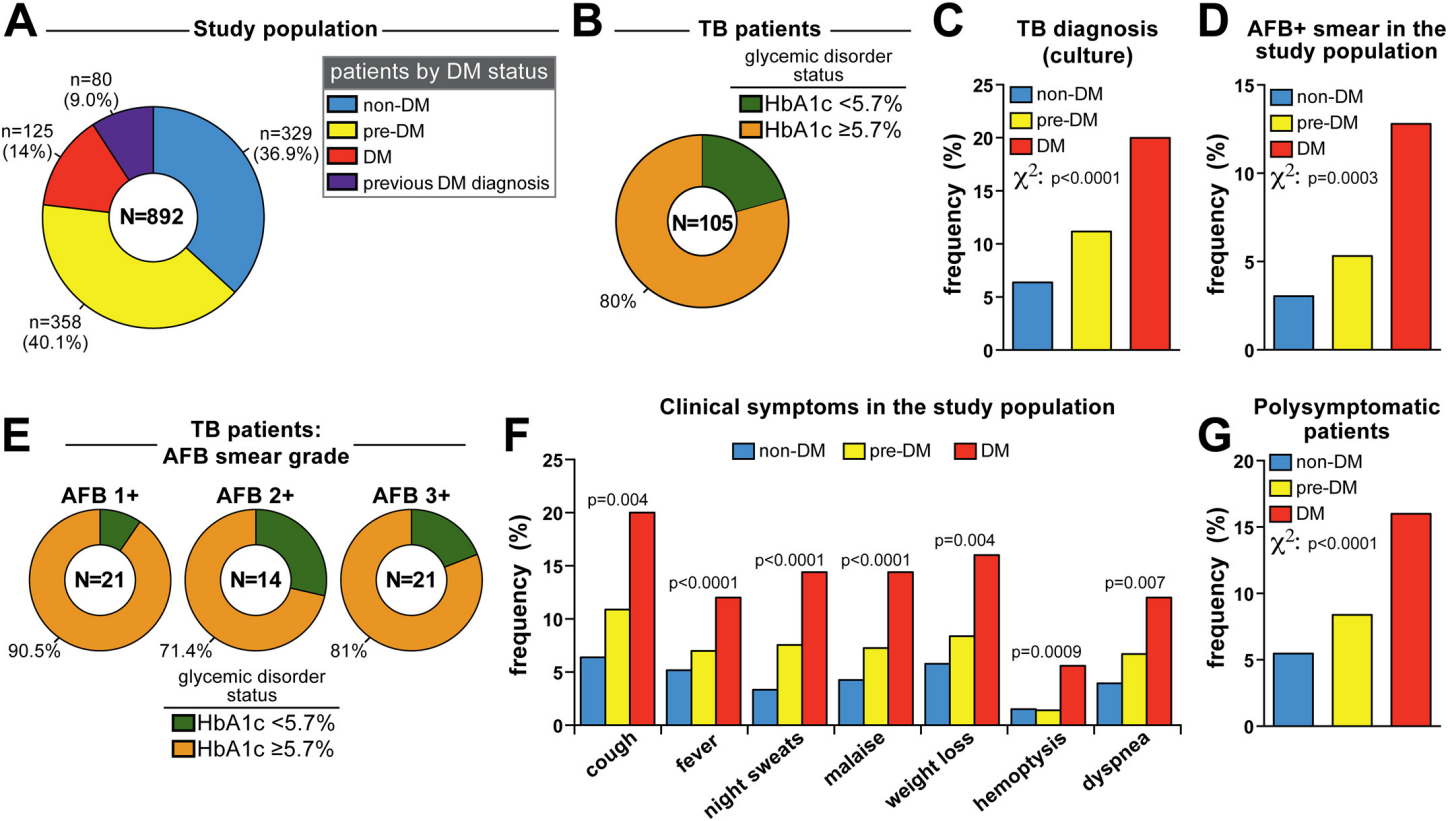
Glucose metabolism disorder is associated with TB in patients with respiratory symptoms. (A) Stratification of patients presenting with respiratory symptoms based on HbA1c levels. (B) Frequency of abnormal HbA1c levels in patients diagnosed with pulmonary TB. (C) Frequency of TB cases in patients without previous DM diagnosis who were classified in non-DM, pre-DM and DM based on Hb1Ac. (D) Frequency of AFB+ in sputum smears from the entire study population. (E) Frequency of TB patients with different AFB smear grades in sputum classified according to altered HbA1c levels. (F) Prevalence of TB-related symptoms relative to the study population was compared between non-DM, pre-DM and DM TB patients without previous DM diagnosis. (G) Individuals were further categorized in polysymptomatic (> 3 symptoms) or non-polysymptomatic (≤ 3 symptoms). In (C), (D), (F) and (G), data were compared using the chi-square test.

Study participants were screened for DM using HbA1c, OGTT and fasting glucose according to ADA guidelines [18]. Notably, 9% (n = 80) of patients self-reported a prior diagnosis of DM. Individuals without prior DM diagnosis (n = 812) were stratified according to HbA1c levels as non-DM (HbA1c <5.7%), pre-DM (HbA1c = 5.7–6.4%) and DM (HbA1c ≥6.5%) (Fig 2A and Table 3). Strikingly, a large majority of patients with respiratory symptoms seeking care at the TB reference clinic exhibited some degree of GMD defined by HbA1c ≥5.7% (n = 563, representing 63.1% of the study population; Fig 2A). These subgroups were similar with regard to gender distribution (female = 57.4% in non-DM vs. 54.2% in pre-DM vs. 53.7% in DM cases; chi-square p = 0.602). Diabetic patients (newly identified and those with previous diagnosis) were on average older than pre-DM and non-DM individuals (Table 3). In addition, median BMI values were higher in diabetics (Table 3). Smoking history was significantly more frequent in individuals with GMD compared with non-DM (Table 3) but the frequency of chronic alcoholism and use of illicit drugs was similar between groups (Table 3). While the prevalence of other comorbidities including HIV/AIDS, cancer, COPD and silicosis was not different between the study groups, the frequency of PTB was higher in patients exhibiting GMD (Table 3 and Fig 2B and 2C). Fully 80% of all PTB cases had HbA1c levels ≥5.7% at the time of diagnosis (Fig 2B), suggesting a potential association between diabetic as well as pre-diabetic GMD with increased odds for TB.

**Table 3:** Characteristics of patients classified according to Hb1Ac levels. Patients with respiratory symptoms (n = 892) were screened for diabetes (DM) and classified as non-DM, pre-DM or DM following the American Diabetes Association criteria (non-DM: HbA1c < 5.7%, pre-DM: HbA1c 5.7–6.4%, DM: referred DM or HbA1c ≥ 6.5%). Frequency data were compared using the chi-square test and continuous variables (age and BMI) were compared using the Kruskal-Wallis test. See Table 1 for abbreviations.

Characteristic	(non-DM /pre-DM /all DM)	non-DM	pre-DM	all DM cases	P-value
		n = 329	n = 358	n = 205	
**Female—no. (%)**	329 /358 /205	189 (57.4)	194 (54.2)	110 (53.7)	0.602
**Median Age—y (IQR)**	329 /358 /205	42 (32–53)	50 (39–58)	56 (48–65)	<0.0001
**Median BMI—Kg/m**^**2**^ **(IQR)**	327 /352 /204	22.2 (19.6–25.0)	22.8 (20.2–27.4)	22.9 (19.7–26.8)	0.026
**Lifestyle habits—no. (%)**					
Chronic Alcoholism	328 /358 /205	14 (4.2)	16 (4.5)	11 (5.4)	0.828
Smoking	328 /357 /205	106 (32.2)	176 (49.3)	96 (46.8)	<0.0001
Illicit drugs use	329 /358 /205	9 (2.7)	10 (2.7)	2 (1.0)	0.332
**Comorbidity—no. (%)**					
Pulmonary TB	329 /358 /205	21 (6.4)	40 (11.2)	44 (21.5)	<0.0001
HIV/AIDS	328 /358 /205	5 (1.5)	3 (0.8)	3 (1.5)	0.680
Cancer	328 /358 /205	3 (0.9)	4 (1.1)	3 (1.5)	0.840
COPD	328 /358 /205	14 (4.3)	12 (3.3)	8 (3.9)	0.823
Silicosis	328 /358 /205	7 (2.1)	11 (3.1)	4 (1.9)	0.628
**Clinical symptoms—no. (%)**					
Cough	329 /358/ 205	21 (6.4)	39 (10.9)	43 (21.0)	0.004
Fever	329 /358/ 205	17 (5.2)	25 (7.0)	26 (12.7)	<0.0001
Night sweats	329 /358/ 205	11 (3.3)	27 (7.5)	32 (15.6)	<0.0001
Malaise	329 /358/ 205	14 (4.2)	26 (7.3)	35 (17.0)	<0.0001
Weight loss	329 /358/ 205	19 (5.8)	30 (8.4)	38 (18.5)	0.004
Hemoptysis	329 /358/ 205	5 (1.5)	5 (1.4)	11 (5.4)	0.0009
Dyspnea	328 /358/ 205	13 (3.9)	24 (6.7)	24 (11.7)	0.007
**AFB status in PTB patients—no. (%)**	21 /40 /44				0.347
Negative		11 (52.4)	21 (52.5)	17 (38.6)	
Positive		10 (47.6)	19 (47.5)	27 (61.4)	

An important parameter related to TB transmission is presence of acid-fast bacilli (AFB) in sputum smears [19]. Positive sputum smears have also been linked to greater TB disease severity and inflammatory profile [20,21]. In the present study, prevalence of AFB+ sputum smear in the study population was significantly higher in newly diagnosed DM cases compared to pre-DM and non-DM individuals (p = 0.0003; Fig 2D). In addition, frequency of individuals presenting altered HbA1c levels was shown to be higher in PTB patients regardless of the AFB status (Fig 2E). We next tested whether non-DM, pre-DM or DM individuals differed in terms of disease presentation based on the prevalence of TB-related symptoms (cough, fever, night sweats, malaise, weight loss, hemoptysis and dyspnea). The frequency of TB-related symptoms increased progressively, following the degree of GMD (Fig 2F). Indeed, newly diagnosed diabetic patients displayed the highest frequency of polysymptomatic individuals compared to the groups of those with pre-DM and non-DM (p <0.0001; Fig 2G). These results reinforce the idea that coincident TB-DM is associated with more severe TB presentation.

**Fig 3:**
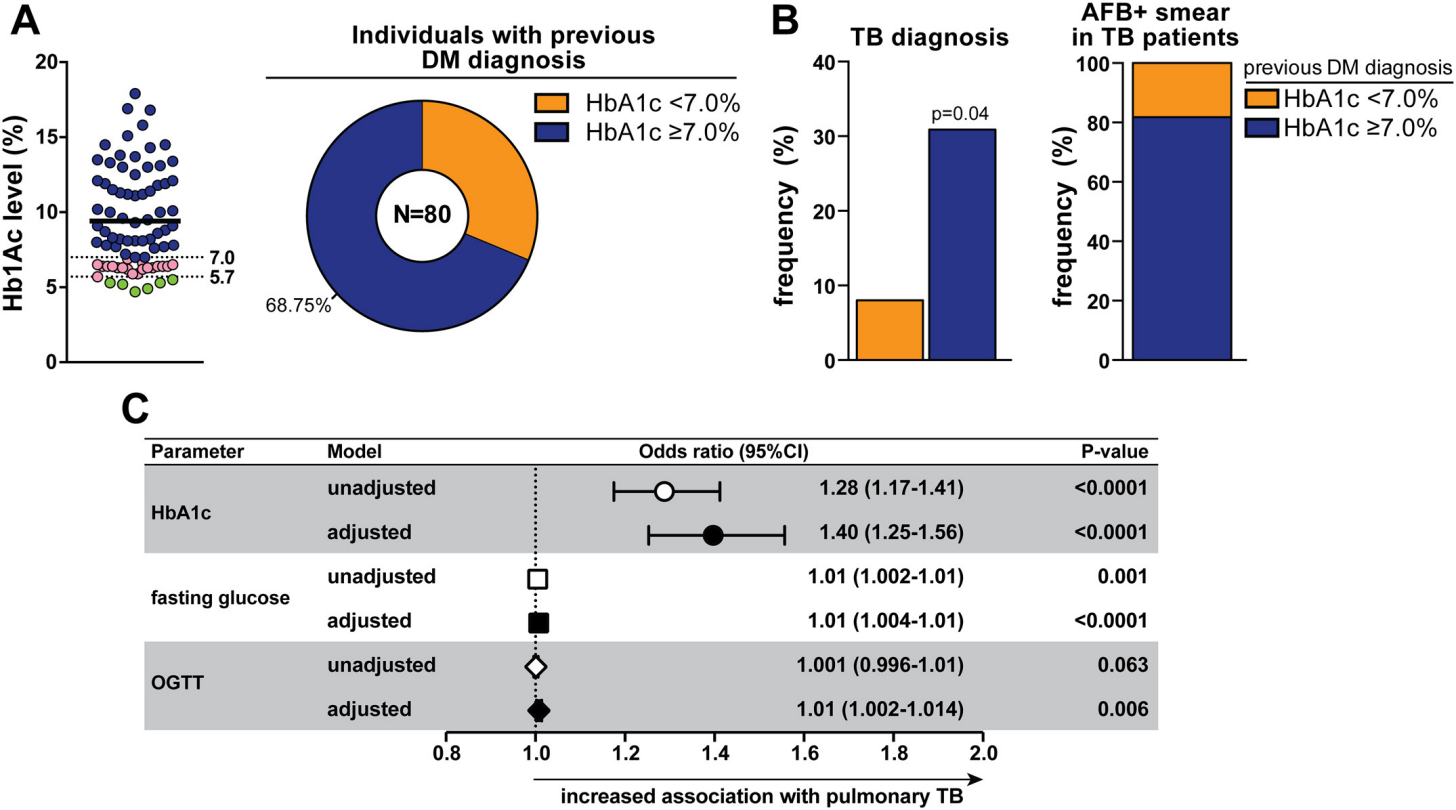
Worse glycemic control is associated with pulmonary TB. (A) Left panel shows Hb1Ac levels in individuals who self reported previous diagnosis of diabetes. Full line represents median value (Hb1Ac = 8.45%). Dotted lines represent thresholds of Hb1Ac used to define normoglycemic levels (5.7%) and uncontrolled DM (7.0%) according to the ADA criteria [18]. Right panel shows frequency of individuals with respiratory symptoms who had referred previous diagnosis of diabetes and presented with poor glycemic control (HbA1c ≥ 7.0%). (B) Frequency of TB diagnosis (left panel) in patients referring previous DM diagnosis, with or without HbA1c ≥ 7.0%. Right panel shows frequency of TB cases with AFB+ sputum smears. The Fisher's exact test was used to assess statistical significance. (C) Linear regression analysis adjusted for age, gender and BMI was used to determine the association between increases of 1 unit in plasma values of HbA1c, fasting glucose or OGTT glycaemia (after log10 transformation) and pulmonary TB in the entire study population (n = 892). The odds associated with the covariates used in the model adjustment are displayed in S1 Table.

Within the total study population, 9% (n = 80) of individuals self-reported prior DM diagnosis. The median HbA1c level in this subpopulation was 8.45% (Fig 3A). Of these patients, six (7.5%) exhibited HbA1c values at normoglycemic level (HbA1c <5.7%) and 15 (18.75%) displayed levels compatible with pre-DM (HbA1c = 5.7–6.4%). Of note, 68.7% (n = 55) of those patients had HbA1c ≥7.0%, indicating poor glycemic control (Fig 3A). Individuals with uncontrolled DM were similar to those with HbA1c <7.0% with regard to age, BMI, gender distribution, lifestyle habits and most of the comorbidities screened (Table 4). Interestingly, the frequency of TB diagnosis was almost 4 times higher in the group of patients with poor glycemic control compared with individuals with HbA1c <7.0% (30.9% vs. 8.0%, respectively, p = 0.026; Table 4 and Fig 3B). Notably, among active TB cases with previous DM diagnosis, individuals with HbA1c ≥7.0% accounted for the majority of AFB+ samples (Fig 3B). Linear regression analysis adjusted for age, gender and BMI confirmed the strong association between heightened values of HbA1c, fasting glucose or OGTT and prevalence of TB in this study population (Fig 3C).

**Table 4:** Characteristics of population with previous diagnosis of diabetes according to the glycemic control status. Patients with respiratory symptoms who referred previous DM diagnosis (n = 80) were classified according to the glycemic control status based on HbA1c levels. Frequency data were compared using the Fisher’s exact test and continuous variables (age and BMI) were compared using the Mann-Whitney test. No patients in these groups referred illicit drugs use. See Table 1 for abbreviations.

Characteristic	(HbA1c <7∙0% / HbA1c ≥ 7∙0%)	HbA1c <7∙0%	HbA1c ≥ 7∙0%	P-value
		n = 25	n = 55	
**Female—no. (%)**	25 /55	11 (44.0)	34 (61.8)	0.152
**Median Age—y (IQR)**	25 /55	63 (54.5–77.0)	58 (49.0–66.0)	0.063
**Median BMI—Kg/m**^**2**^ **(IQR)**	24 /55	24.9(16.6–26.0)	22.5 (19.5–26.1)	0.359
**Lifestyle habits—no. (%)**				
Chronic Alcoholism	25 /55	2 (8.0)	2 (3.6)	0.585
Smoking	25 /55	13 (52.0)	19 (34.5)	0.462
**Comorbidity—no. (%)**				
Pulmonary TB	23 /52	2 (8.7)	17 (32.7)	0.042
HIV/AIDS	25 /55	1 (4.0)	1 (1.8)	0.530
Cancer	25 /55	2 (8.0)	0 (0)	0.094
COPD	25 /55	1 (4.0)	1 (1.8)	0.530
Silicosis	25 /55	0 (0)	3 (5.4)	0.548
**AFB status in PTB patients—no. (%)**	02 /17			0.485
negative		0 (0)	8 (47)	
positive		2 (100)	9 (53)	

**Fig 4:**
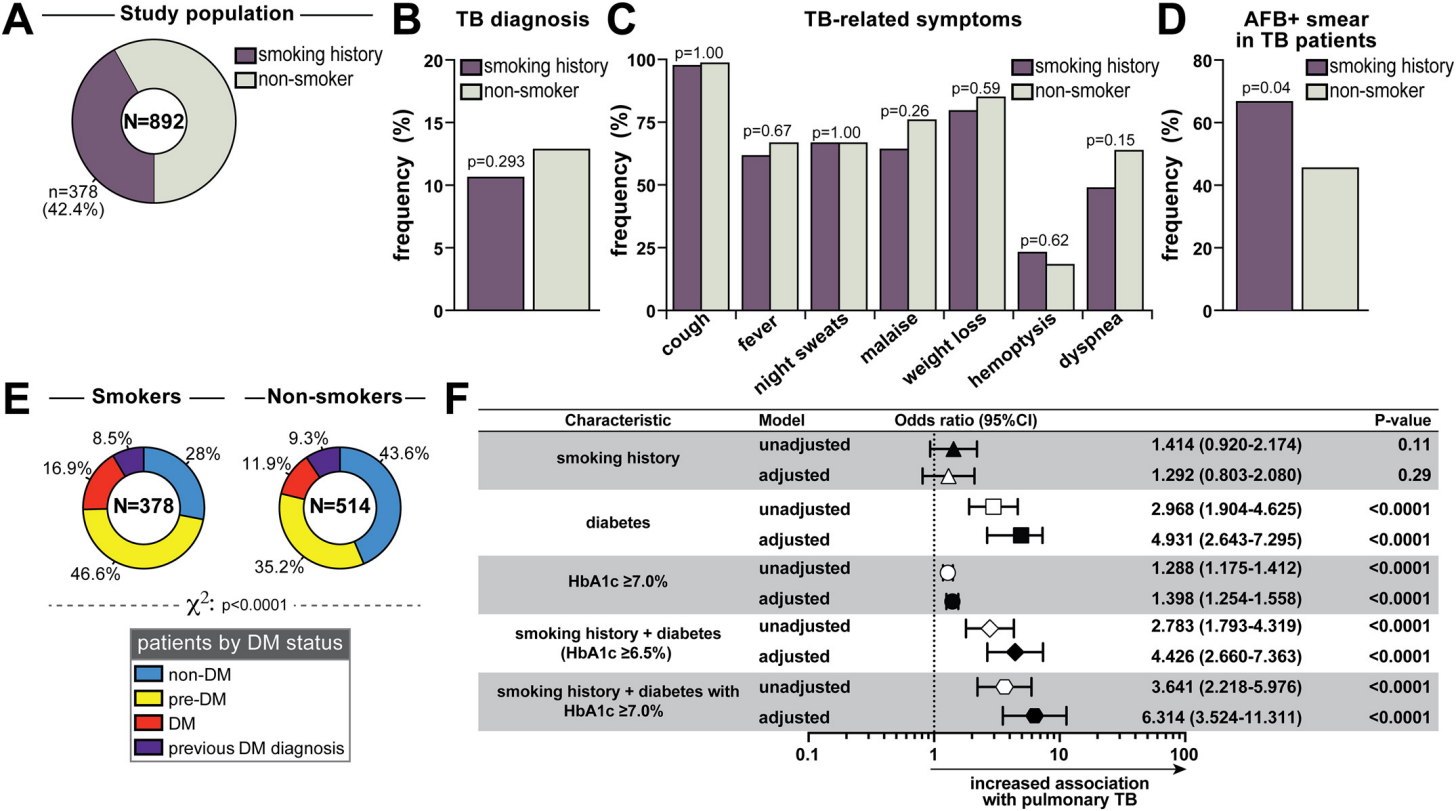
Associations between cigarette smoking, glycemic control and pulmonary tuberculosis. (A) Frequency of smokers in the entire study population. (B) Frequency of TB diagnosis in smoking and non-smoking individuals with respiratory symptoms. (C) Prevalence of TB-related symptoms was compared between smoking and non-smoking PTB patients. (D) Frequency of AFB+ in sputum smears from smoking and non-smoking TB patients. In (B), (C) and (D), data were compared using the Fisher’s exact test. (E) Frequency of non-DM, pre-DM, DM (stratified according to HbA1c values) and those who referred previous diagnosis of DM in the groups of smoking or non-smoking individuals. Data were compared using the chi-square test. (F) Multinomial regression analysis adjusted for age, gender and BMI, was perform to test the association of the indicated conditions and pulmonary TB. The odds associated with the covariates used in the model adjustment are displayed in S1 Table.

Cigarette smoking is an independent risk factor for pulmonary TB [3]. A prospective 14-year cohort study of over 1.2 million South Koreans showed that smoking was associated with increased mortality from TB in men and women, with similar risks for current and former smokers [22]. In our study, 42.4% of the subjects reported smoking history (Table 5 and Fig 4A). Female gender was more prevalent in the group of non-smokers (Table 5). Former smokers were older (Table 5). BMI was higher in non-smokers, while current smokers had more deleterious life style habits than the other groups (Table 5). Frequency of HIV/AIDS, cancer, COPD and silicosis was not different between the groups stratified according to smoking history (Table 5). Contrasting with data from the Korean population^20^, the prevalence of pulmonary TB among our Brazilian cohort was not increased in subjects with smoking history compared to non-smokers (12.4% in current smokers vs. 9.2% in former smokers vs. 12.8% in non-smokers, p = 0.359; Table 5). Even when all patients with any smoking history were pooled in a single group, we could not detect a significant difference in TB prevalence compared with non-smokers (p = 0.293; Fig 4B). Furthermore, within the group of PTB patients (n = 105), the frequency of TB-related symptoms was not different between non-smoking individuals and those with smoking history (Fig 4C), suggesting that smoking did not impact the disease presentation profile in our study population. Nevertheless, we detected a significantly increased incidence of AFB+ sputum smears in smokers compared with non-smokers (p = 0.044; Fig 4D). The combination of smoking and DM has been described to cause a synergistic effect on the risk of death within 12 months of TB diagnosis [23,24]. In the present study, the prevalence of GMD was substantially higher in the group of individuals referring smoking history than in non-smokers (p <0.0001; Fig 4E). Finally, we performed multinomial logistic regression analysis adjusted for age, gender and BMI to assess direct associations between smoking history, DM or the combination of these two risk factors and incidence of TB diagnosis. We found that smoking history alone was not significantly associated with increased odds for PTB (adjusted OR: 1.2, 95% CI: 0.80–2.08, p = 0.291; Fig 4F). As expected by our preliminary analyses, DM was significantly associated with TB (Fig 4F). The strength of association was even higher when only uncontrolled DM was considered (HbA1c ≥7.0%) (Fig 4F). When smoking history and DM were considered together, we observed a significant association with PTB (adjusted OR: 4.4, 95% CI: 2.7–7.4, p <0.0001; Fig 4F). Importantly, individuals with combined smoking history and uncontrolled DM (HbA1c ≥7.0%) exhibited 6 times greater odds for PTB diagnosis than those without these risk factors (adjusted OR: 6.3, 95% CI: 3.5–11.3, p <0.0001; Fig 4F).

**Table 5:** Characteristics of population according to smoking status. Patients with respiratory symptoms (n = 892) were classified as current smokers, former smokers and non-smokers. Frequency data were compared using Chi-square test and continuous variables (age and BMI) were compared using the Kruskal-Wallis test. See Table 1 for abbreviations.

Characteristic	(current smoker /former smoker /non-smoker)	Current smoker	Former smoker	Non-smokers	P-value
		n = 140	n = 238	n = 514	
**Female—no. (%)**	140 /238 /514	59 (42.1)	109 (45.8)	325 (63.2)	<0.0001
**Median Age—y (IQR)**	140 /238 /514	51.5 (41–58)	54.5 (46–63)	45 (34.0–55.0)	<0.0001
**Median BMI—Kg/m**^**2**^ **(IQR)**	138 /234 /509	20.66 (18.9–23.6)	22.82 (20.4–27.4)	23.2 (20.1–26.6)	<0.0001
**Lifestyle habits—no. (%)**					
Chronic Alcoholism	140 /238 /513	20 (14.3)	7 (2.9)	14 (2.7)	<0.0001
Illicit drugs use	140 /238 /513	15 (10.7)	6 (2.5)	0 (0)	<0.0001
**Comorbidity—no. (%)**					
Pulmonary TB	140 /238 /514	17 (12.1)	22 (9.2)	66 (12.8)	0.359
HIV/AIDS	140 /238 /514	4 (2.9)	3 (1.3)	4 (0.8)	0.141
Cancer	140 /238 /514	0 (0)	7 (2.9)	3 (0.6)	0.006
COPD	140 /238 /514	6 (4.3)	13 (5.5)	15 (2.9)	0.226
Silicosis	140 /238 /514	4 (2.9)	11 (4.6)	7 (1.4)	0.026
**Clinical symptoms in TB patients- no. (%)**					
cough	17 /22 /66	17 (100)	21 (95.4)	65 (98.5)	0.547
fever	17 /22 /66	11 (64.7)	13 (59.1)	44 (66.7)	0.812
night sweats	17 /22 /66	12 (70.6)	14 (63.6)	44 (66.7)	0.901
malaise	17 /22 /66	10 (58.8)	15 (68.2)	50 (75.7)	0.360
weight loss	17 /22 /66	13 (76.5)	18 (81.8)	56 (84.8)	0.708
hemoptysis	17 /22 /66	4 (23.5)	5 (22.7)	12 (18.2)	0.830
dyspnea	17 /22 /66	6 (35.3)	13 (59.1)	42 (63.6)	0.106
**AFB status in PTB patients—no. (%)**	17 /22 /66				0.060
negative		4 (24)	9 (41)	36 (54)	
positive		13 (76)	13 (59)	30 (46)	

## Discussion

A relationship between DM and TB has been appreciated for several years [8] but has only lately been a focus of research. Worldwide DM has increased by ~20% in the past three decades, high with the greatest increase in low and middle-income countries [25]. In these countries, where TB is among the primary causes of death, this dual burden leads to substantial impact on public health [5]. Reinforcing this idea, we have recently shown that TBDM patients exhibit worse clinical TB presentation and adverse outcomes compared to non-DM TB individuals from Brazil [14]. The present study examined the interaction between plasma glucose levels and PTB in a TB-endemic area of Brazil. We found a high prevalence of GMD in patients presenting with respiratory symptoms at a TB primary care center. Whether TB promotes GMD in susceptible people or if GMD directly affects TB susceptibility is incompletely understood but both effects are likely and each has important implications for public health. A preponderance of evidence suggests that DM impairs human immunity to TB, increasing the risk for progression of latent TB infection and the severity of TB disease [8,26]. Results from our study and others, suggest that a broader spectrum of GMD, including pre-DM, may have a similar effect [27,28]. If this were true, then the TB burden attributable to metabolic syndromes would be far greater than is currently estimated for DM alone. Protective immunity against TB might also be affected by glucose-independent pathways. This concept is supported by evidence that pre-diabetic GMD in non-diabetic individuals is associated with increased risk vascular complications [29,30]. An alternative, but not mutually exclusive hypothesis is that the inflammatory stress of active TB leads to insulin resistance and/or reduced beta cell function, thereby promoting GMD (or deteriorating DM disease) with implications for bidirectional screening and for DM prevention [31,32].

The majority (63.1%) in our cohort of patients with respiratory symptoms exhibited some degree of GMD. Patients with GMD in our cohort not only had higher prevalence of TB but were also more likely to have TB-related symptoms. These results argue that GMD affects TB disease severity. This is consistent with other studies reporting that comorbid DM increases the odds of positive sputum smear, cavitation, delayed sputum conversion, treatment failure and recurrent TB [8,12,28], all of which increase the risk of transmission to the general population. In addition, we found that up to 69% of the individuals self-reporting previous DM diagnosis had HbA1c ≥ 7.0%, indicating poor glycemic control. Notably, TB prevalence was higher in individuals with HbA1c ≥ 7.0% compared with those with controlled DM, reinforcing the association between poor glycemic control and TB [30]. Regression analyses adjusted for age, gender and BMI demonstrated that increased plasma values of HbA1c, fasting glucose or OGTT glycaemia were all independently associated with TB in symptomatic respiratory patients, even those falling below the ADA cutoff for DM.

In the present study we found an increased prevalence of GMD in patients with a history of smoking. Prospective studies from South Korea concluded that smoking increases risk of incidence, mortality and recurrence of TB [22,24]. In our Brazilian cohort, where 42.4% of individuals were past or current smokers, there was no statistically significant association between smoking and incident TB. In addition, we found no difference in frequency of TB-related symptoms in smokers vs. nonsmokers. Nevertheless, in patients diagnosed with TB, smoking history was associated with higher frequency of positive sputum AFB smear. The lack of association between smoking and incident TB observed here might be attributable to an enrichment of female patients in our cohort, since a Korean study found that the association of TB with smoking was limited to male gender [22]. Another Korean cohort study reported that the combination of smoking and DM have a synergistic effect on all-cause mortality and TB-related mortality within 12 months of ATT initiation, after adjusting for other confounders [24]. Multinomial logistic regression analyses of data from our Brazilian cohort confirmed this synergistic effect of smoking and uncontrolled DM (HbA1c ≥7.0%) on increased association with TB. The mechanisms driving this association remain unknown and merit further investigation.

Our data shows no difference in COPD prevalence with respect to smoking history. The association between COPD and smoking is dependent on the number of cigarettes and frequency of smoking. We did not have information on either of these parameters. It is possible that the tobacco burden (pack-years) was low in this population. In addition, the overall frequency of COPD in our study population was low (34 out of 892 individuals). It is possible that the overall low frequency of COPD could have reduced the power to detect differences induced by smoking. Lastly, documentation of COPD was based on self-report, and we did not employ spirometric assays to diagnose this condition. Importantly, there is published evidence for COPD in non-smokers, which is reported at different rates in different countries and populations with the lowest rate in developed nations with good air quality and industrial hygiene enforcement and highest rates in countries with the worst air pollution and biomass combustion [33].

Our study has several limitations. Our study lacked systematic evaluation of radiographic data on TB disease. We thus were unable to analyze prevalence of cavitary lesions and extent of lung disease (unilateral/bilateral) between the study groups. A previous study from our group performed in the same TB reference center revealed that occurrence of cavitation in TB patients was not affected by DM [14]. We did not have access to information regarding DM severity (time since diagnosis and the presence of DM-related complications). Nor was there data on other metabolic comorbidities such as dyslipidemia or vitamin D insufficiency that have increased prevalence in DM patients and may contribute to TB susceptibility. Our conclusions are also limited by lack of information on treatment response, TB outcomes and glycemic status at the completion of ATT. Additional prospective studies are needed to define the individual and cumulative impact of metabolic disorders and lifestyle habits on TB susceptibility and outcomes and the impact of TB on glycemic status.

In summary, our data indicate that sustained diabetic hyperglycemia, as well as pre-diabetic GMD, increases the risk for PTB in Brazilian patients with respiratory symptoms. We further found an adverse combinatorial effect of GMD and smoking on TB risk in this population. In such epidemiological settings, bidirectional screening and integrated management may help to improve early diagnosis and clinical outcomes for both TB and DM.

## Supporting Information

S1 Table Association between covariates and odds for pulmonary TB in the study population. (DOCX)
