# Supplementary material for: Glucose Metabolism Disorder Is Associated with Pulmonary Tuberculosis in Individuals with Respiratory Symptoms from Brazil
Source: PLoS One. 2016 Apr 14;11(4):e0153590. doi: 10.1371/journal.pone.0153590 (PMC4831681; doi:10.1371/journal.pone.0153590)
Supplement: S1 Table — (DOCX) [file pone.0153590.s001.docx]

**S1 Table. Association between covariates and odds for pulmonary TB in the study population**

| **Covariates** | **Odds Ratio (95%CI)** | **P-value** |
| --- | --- | --- |
| Male gender | 1.369 (0.90-2.082) | 0.142 |
| Age | 1.031 (1.016-1.047) | <0.0001 |
| BMI | 1.161 (1.097-1.229) | <0.0001 |

Univariate associations between covariates and odds for pulmonar TB. For male gender, nominal regression was employed whereas for age and BMI, odds are for increases in 1 unit using binary logistic regression analysis.
